# Investigating Verticillium wilt occurrence in cotton and its risk management by the direct return of cotton plants infected with *Verticillium dahliae* to the field

**DOI:** 10.3389/fpls.2023.1220921

**Published:** 2023-11-03

**Authors:** Guangjie Zhang, Zhuo Meng, Hao Ge, Jiali Yuan, Song Qiang, Ping’an Jiang, Deying Ma

**Affiliations:** ^1^ College of Agronomy, Xinjiang Agricultural University, Urumqi, China; ^2^ Engineering Research Centre of Cotton, Ministry of Education, Xinjiang Agricultural University, Urumqi, China; ^3^ Key Laboratory of the Pest Monitoring and Safety Control on Crop and Forest, Xinjiang Agricultural University, Urumqi, China

**Keywords:** stalks returning to field, cotton Verticillium wilt, growth and development, biotransformation, green prevention and control

## Abstract

Verticillium wilt is one of the most crucial diseases caused by *Verticillium dahliae* that threatens the cotton industry. Statistical results showed that the return of cotton plants infected with *V. dahliae* to the field might be an essential cause of the continuous aggravation of cotton Verticillium wilt. The correlation among the cotton plants infected with *V. dahliae* returning to the field, the occurrence of Verticillium wilt, and the number of microsclerotia in rhizosphere soil need further investigation. A potted experiment was carried out to explore the effects of the direct return of cotton plants infected with *Verticillium dahliae* to the field on the subsequent growth and Verticillium wilt occurrence in cotton. As a risk response plan, we investigated the feasibility of returning dung-sand (i.e., insect excreta) to the field, the dung-sand was from the larvae of *Protaetia brevitarsis* (Coleoptera: Cetoniidea) that were fed with the *V. dahliae*–infected cotton plants. The results demonstrated that the return of the entire cotton plants to the field presented a promotional effect on the growth and development of cotton, whereas the return of a single root stubble or cotton stalks had an inhibitive effect. The return of cotton stalks and root stubble infected with *V. dahliae* increased the risk and degree of Verticillium wilt occurrence. The disease index of Verticillium wilt occurrence in cotton was positively correlated with the number of microsclerotia in the rhizosphere soil. The disease index increased by 20.00%, and the number of soil microsclerotia increased by 8.37 fold in the treatment of returning root stubble infected with *V. dahliae* to the field. No Verticillium wilt microsclerotia were detected in the feed prepared from cotton stalks and root stubble fermented for more than 5 days or in the transformed dung-sand. There was no risk of inoculation with Verticillium wilt microsclerotia when the dung-sand was returned to the field. The indirect return of cotton plants infected with *V. dahliae* to the field by microorganism–insect systems is worthy of further exploration plan of the green prevention and control for Verticillium wilt and the sustainable development of the cotton industry.

## 1 Introduction

Xinjiang Uygur Autonomous Region is the largest cotton (*Gossypium hirsutum* L.)–growing region in China, with more than 2.5 million hectares planted and more than 5 million tons of cotton produced (Announcement of the National Bureau of Statistics, 2022), accounting for about 20% of the world’s cotton production (Wang et al., 2022). Because of limited land and water resources in northwest China, cotton fields that have been continuously cropped for over 20 years are prevalent in the Xinjiang cotton region (Wang et al., 2012). Cotton plants are rich in organic matter and nutrients. The direct return of cotton plants to the field has been carried out in Xinjiang for more than 40 years. Under the premise of proper operation, returning cotton plants to the field can promote the growth and development and yield of cotton (Wang et al., 2014; Lu et al., 2022; Xi et al., 2022; Yan et al., 2022; Zhang et al., 2023a). For instance, treatments with straw and straw biochar significantly enhanced most cotton growth parameters, consequently increasing the seed cotton yield from the second to the fourth year (Ma et al., 2019). Straw retention coupled with mineral phosphorus fertilizer reduces phosphorus fertilizer input and improves cotton yield in coastal saline soils (Cao et al., 2021). Straw returning coupled with nitrogen fertilization increases canopy photosynthetic capacity, yield, and nitrogen use efficiency in cotton (Hu et al., 2021). On the contrary, there are also negative reports on the growth and yield of cotton (Soon and Lupwayi, 2012). Verticillium wilt is a worldwide soil-borne fungal disease caused by *Verticillium dahliae* (Hampton et al., 1990; Eldon and Hillocks, 1996; Wang et al., 2004). It has become one of the crucial diseases threatening the sustainable development of the cotton industry and has shown an increasing trend year after year (Wang et al., 2011; Zhu et al., 2017; Liu et al., 2019). Microsclerotia are the primary survival structures in *V. dahliae* in soil and the primary infection source of cotton Verticillium wilt. Because of their strong adaptability, microsclerotia can survive in the soil for more than 10 years and are widely distributed in soil (Wilhelm, 1955; Devay et al., 1974). Studies have shown that the number of microsclerotia in rhizosphere soil is closely related to the occurrence of Verticillium wilt in crops (Harris and Yang, 1996; Barbara et al., 2007; López-Escudero and Blanco-López, 2007). Statistical findings from previous studies on cotton Verticillium wilt conducted across diverse regions and years of continuous cropping have consistently demonstrated that the re-introduction of *V. dahliae*–infected cotton plants into the field in successive years constitutes a significant contributing factor to the progressive exacerbation of cotton Verticillium wilt (Pullman and Devay, 1982; Paplomatas, 1992; Gao and Guo, 2020). However, the effects of returning cotton plants infected with *V. dahliae* to the field on the occurrence of Verticillium wilt and the number of microsclerotia of cotton in the next or current years remain unclear (Liu et al., 2018a; Luo et al., 2018).

The most effective method for preventing and controlling Verticillium wilt is breeding for disease resistance, but the progress could be faster. No cotton varieties have high resistance to cotton Verticillium wilt, and the chemical control is poorly effective and causes environmental pollution (Zhu et al., 2017). Biological control is considered the most ideal for controlling cotton Verticillium wilt. For instance, many studies have proven the biological control effect against verticillium wilt to be above 50% in greenhouse experiments; some studies have gradually applied it to the field, but its effectiveness in the field is not yet satisfactory (Yuan, 2006; Zheng et al., 2011; Lang et al., 2012; Li et al., 2013; Li et al., 2014; Wei et al., 2019a; Zhang et al., 2021). It is worth noting that the cultivation system of cotton plants returning directly to the field is closely related to the occurrence of cotton Verticillium wilt (Gao and Guo, 2020; Zhang et al., 2023b). How to properly make good use of cotton plants in diseased fields and realize green prevention and control of cotton Verticillium wilt is a problem worthy of further study (Wei et al., 2019b; Wheeler et al., 2019; Wheeler et al., 2020; Liu et al., 2021a). As a risk response plan, we plan to evaluate the feasibility of returning dung-sand (insect excreta, where the more prominent grain is called dung-sand) to the field, and the dung-sand was from the larvae of *Protaetia brevitarsis* Lewis (Coleoptera: Cetoniidea) that were fed with the *V. dahliae*–infected cotton plants.

*P. brevitarsis* is an insect belonging to the genus *Protaetia*, family Cetoniidae, and order Coleoptera (Ma, 1995; Ji et al., 2011). The larvae that are saprovorous and can efficiently break down crop stalks (Liu and Zhang, 2015; Yang et al., 2015; Zhang et al., 2019), livestock and poultry manure (Guo, 2020; Xu et al., 2021; Yang et al., 2022), edible fungus chaff (Zhang, 2015; Lee et al., 2018; Sun, 2018; Wei et al., 2020), and other organic wastes that decomposed microorganisms have pretreated. In particular, the larvae have an outstanding transformation ability for cotton stalks and can yield high-value products (Zhang et al., 2022). The larvae have the potential for application in feedingization (Lee et al., 2020; Ham et al., 2021; Nikkhah et al., 2021). Larvae dung-sand can be applied as fertilizer (Zhang et al., 2020). Previous studies have shown that larvae dung-sand is rich in humic acids and nutrient elements (Li et al., 2019) and has just begun to be used as an organic fertilizer or cultivation substrate for horticultural crops (Liu et al., 2018b; Lai et al., 2019; Wu et al., 2019; Shi et al., 2021; Joung et al., 2022), and the digestive system of the larvae of *P. brevitarsis* can kill plant pathogenic microorganisms (Xuan et al., 2022). However, there have been no studies on the transformation ability of the larvae of *P. brevitarsis* on the cotton stalks (aboveground part of the cotton plants) and root stubble (aboveground stubble of 10 cm + the underground root) infected with *V. dahliae*. The effects of decomposed microorganism and insect systems on the number of Verticillium wilt microsclerotia and the risk of returning dung-sand, which was from the larvae of *P. brevitarsis* fed with cotton plants infected with *V. dahliae* to the field are not known.

In summary, in the context of the large-scale direct return of cotton plants infected with *V. dahliae* to the field and the increasingly severe occurrence of Verticillium wilt in Xinjiang, it is essential to study the effects of these phenomena on cotton growth and development; in addition, the occurrence of Verticillium wilt and the number of microsclerotia in rhizosphere soil is worthy of further discussion. The feasibility of returning cotton plants infected with *V. dahliae* after transformation by larvae dung-sand is also worthy of further investigation. This study can provide data and reference schemes for the recycling of cotton plants infected with *V. dahliae* and the green control of cotton Verticillium wilt.

## 2 Materials and methods

### 2.1 Test site

The test site was located in the Industrialization Research Base of Environmental Insect Transforming Organic Waste, Xinjiang Agricultural University, in Manas County (44°13′49″ N, 86°23′3″ E), Changji Prefecture, China.

### 2.2 Experimental materials

Cotton plants and potted soil were obtained from cotton Verticillium wilt fields around the test site. Cotton Verticillium wilt inoculants (wheat grains overgrown with *V. dahliae*) were purchased from the Cotton Research Institute of the Chinese Academy of Agricultural Sciences (Anyang, China); the strain type was common Verticillium wilt species (sclerotium type and deciduous type; the effective number of viable *V. dahliae* was 1.0 × 10^8^ g^−1^) in the cotton fields of Xinjiang region. The cotton variety was Xinluzao 67 (Xinjiang Yanshi Dehai Agricultural Science and Technology Co., Ltd., Tiemengguan, China). The decomposition inoculant was VT (VT-1000, main functional bacteria are *Bacillus*, actinomycetes, lactic acid bacteria, and molds; effective number of viable bacteria was 2.0 × 10^10^ g^−1^; Beijing VOTO Biotechnology Co., Ltd., Beijing, China). Autoclave (vertical pressure steam sterilizer, YXC.5CS.1, Shanghai Bosun Medical Biological Instrument Co., Ltd., Shanghai, China), an instrument to measure chlorophyll (SPAD-502plus, Konica Minolta, Tokyo, Japan), electronic balance (LT3002, Changshu Tianliang Instrument Co., Ltd., Changshu, China), and vernier caliper (Hugong, 1–150 mm; Shanghai, China) were owned by the base. Flower pots (plastic material; the bottom has six small holes with a diameter of 1 cm, the upper and lower diameters are 30 cm and 24 cm, respectively; and the height is 30 cm), cotton seeds, tape, and other materials were purchased from the Shihezi farmers market. The larvae of *P. brevitarsis* were reproduced in the base.

### 2.3 Experimental methods

#### 2.3.1 Effects of the direct return of cotton stalks and root stubble infected with *Verticillium dahliae* to the field on cotton growth and development

In advance, cotton plants infected with *V. dahliae* were divided into two parts: cotton stalks (the aboveground part of the cotton plants) and root stubble (the aboveground stubble of 10 cm + the underground root) (when cotton plants were mechanically harvested, all cotton leaves and a portion of cotton branches detached from the cotton plants, allowing for the harvest of dry weight of both cotton stalks and root stubble in a 1:1 ratio). The cotton stalks and root stubble were chopped into smaller pieces measuring less than 2 cm in length. The cotton stalks, root stubble, and cotton plants (half cotton stalks and half root stubble) were returned to the field with weights of 300 g m^−2^, 300 g m^−2^, and 600 g m^−2^ (the total collected amount of cotton plants was 6000 kg hm^−2^). The design of the potted experiment is shown in **Table 1**. Cotton stalks or root stubble infected with *V. dahliae* were inoculated with Verticillium wilt inoculants at a rate of 6‰ of dry weight, whereas diseased soil was inoculated at a rate of 45 g m^−2^ (part of the samples was reserved for the determination of Verticillium wilt microsclerotia). Then, half of the potting substrate and additives was sterilized in the autoclave (121°C, 20 min; to ensure that *V. dahliae* is completely inactivated). The dry weight of each treated potted substrate was 10 kg (up to 25-cm deep). During the test, cultured pots were buried in the experimental field (the experimental field had not been planted with cotton in recent years) while keeping the top edge of the pots 2 cm above the ground (the purpose of this arrangement was to facilitate temperature and humidity to remain stable and reduce environmental interference). Two-meter-wide corridors separated the planting plots belonging to the diseased and sterilized experiment. For each treatment, 12 repeats were set up with control (CK) in the middle of the first six treatments; the layout was from north to south with six columns with aisle spacing of 60 cm. The additives or Verticillium wilt inoculants were uniformly returned to the top 15 cm of the soil before sowing. Four cotton seeds were planted in each pot and watered after sowing. After seedling emergence, one strong seedling was retained, and uniform management practices such as watering (2 L of water every 7 days), weeding (during the cotton seedling and bud stage), and pest control (for aphids and leaf mites) were implemented for all pots. The plant height, stem diameter, leaf age, height of the first fruiting branch, the number of fruiting branches, and chlorophyll content [Soil and plant analyzer development (SPAD) value and inverted three leaves] were investigated in each treatment and control at 30, 50, and 70 days, respectively.

**Table 1:** Experimental design to study the effects of returning cotton plants infected with *V. dahliae* to the field on the growth and development of cotton and the occurrence of Verticillium wilt.

Treatments	Potting substrate	Additives
1	Sterilized diseased field soil	Cotton stalks infected with *V. dahliae*
2		Root stubble infected with *V. dahliae*
3		Cotton plants infected with *V. dahliae*
4		Sterilized cotton stalks
5		Sterilized root stubble
6		Sterilized cotton plants
CK1		None
7	Diseased field soil with *V. dahliae*	Cotton stalks infected with *V. dahliae*
8		Root stubble infected with *V. dahliae*
9		Cotton plants infected with *V. dahliae*
10		Sterilized cotton stalks
11		Sterilized root stubble
12		Sterilized cotton plants
CK2		None

#### 2.3.2 Effects of directly returning cotton stalks and root stubble infected with *V. dahliae* to the field on the occurrence of cotton Verticillium wilt and the number of microsclerotia in the rhizosphere soil

After allowing potted cotton to grow for 40 days, the grade of cotton Verticillium wilt was investigated (according to the five-grade classification described in GBT22101.5-2009, China); grading was then performed every 10 days. The average disease index of each of the four potted cotton plants was considered as one replicate, which was repeated thrice to calculate the disease index of each treatment. The equation to do so was as follows:


(1) $$\begin{array}{l} Disease\;index=[\Sigma (number\;of\;diseased\;plants\;at\;all\;levels\times correponding\;disease\;grade)/\\ total\;number\;of\;plants\;investigated\times highest\;disease\;grade\;(4)]\times 100 \end{array}$$

After 70 days of growing potted cotton, samples of the rhizosphere soil from treatments 1–3 and 7–12, CK1, and CK2 (collected from 10 cm~15 cm below the surface, four pots as one replicate, repeated thrice) were collected. The number of Verticillium wilt microsclerotia was determined in the experimental and initial samples {the detection method was adapted from that of Wei et al. (2015) and was based on molecular biological detection methods [Synergy Brands (SYBR) Green real-time quantitative polymerase chain reaction of wet-sieving samples (wet-sieving Quantitative real time polymerase chain reaction (qPCR))] performed at the detection institution at the Cotton Research Institute, Chinese Academy of Agricultural Sciences, Anyang, China}. The aim of this study was to investigate the effects of the direct return of cotton stalks and root stubble infected with *V. dahliae* to the field on the occurrence of cotton Verticillium wilt and the number of microsclerotia in rhizosphere soil.

#### 2.3.3 Transformation ability of insect–microorganism composite technology on the cotton stalks and root stubble with *V. dahliae* and risk assessment of this system for the spreading of cotton Verticillium wilt

Silage machinery was used to harvest cotton stalks from the severely diseased fields where Verticillium wilt occurred. The lifting machinery pulled out the corresponding root stubble. A guillotine grinder crushed cotton stalks and root stubble, and, then, 60 kg (dry weight) of each was sterilized by the autoclave (121°C, 20 min). To ensure the adequacy and uniformity of the number of microsclerotia present after crushing the two materials, 160 kg of the cotton stalks and root stubble, according to 6‰ of dry weight of materials, was added the cotton Verticillium wilt inoculants, followed by adjustment of the water content to 65% ± 5%. The resulting materials were cultured at room temperature for 5 days.

The four mixed materials finally obtained were 60 kg of sterilized cotton stalks, sterilized root stubble, cotton stalks with *V. dahliae*, and root stubble with *V. dahliae*. These were supplemented with 40 kg of cattle manure (the main feed was wheat stalks, and no microsclerotia of cotton Verticillium wilt was detected) and 1‰ VT. The two controls contained 100 kg of cotton stalks infected with *V. dahliae* and root stubble with *V. dahliae*. The fermentation of materials (duration of 25 days) and the transformation of the larvae of *P. brevitarsis* were carried out according to the methodology of Zhang et al. (2022) [each culture box (1 L) was filled with 280 g of fresh material (about 80 g dry weight), 10 larvae (the third instar and 15th day) of *P. brevitarsis* were put into the box. The transformation experiment was carried out for 15 days. Each treatment was repeated four times]. After the transformation, we determined the weight gain, feed intake, and the yield of larvae dung-sand. In addition, the feed utilization rate, dung-sand conversion rate, and mortality were calculated with the method of Liu (2012). The equation used for calculation was as follows (mass unit = mg):


(2) Feed utilization rate (FUR)=(total feed weight−remaining feed weight)/total feed weight×100%

(3) Dung-sand conversion rate=Dung-sand weight/(feeding weight−dry larvae weight gain)×100%

(4) Mortality=Number of dead larvae/number of testes larvae×100%

To evaluate the risk of the spread of cotton Verticillium wilt by the insect–microorganism composite technology, samples were collected at 0, 5, 10, 15, 20, and 25 days during the fermentation process of the six kinds of materials, respectively. The number of cotton Verticillium wilt microsclerotia was determined in these materials and the dung-sand from *P. brevitarsis* larvae that had been fed the six kinds of materials after fermenting it for 25 days (the specific detection method was referred to in Section 2.3.3). This section focuses on evaluating the effects of the fermentation process and the application of the insect–microorganism composite technology.

### 2.4 Data processing

SPSS 23.0 was used to perform one-way analysis of variance and the Tukey’s multiple comparisons analysis to quantify the differences among the treatments (*P<* 0.05). Microsoft Excel 2013 was used to record and organize data and draw tables; Sigma Plot 14 was used to draw graphs.

## 3 Results

### 3.1 Effects of the direct return of cotton stalks and root stubble infected with *Verticillium dahliae* to the field on cotton growth and development

After being allowed to grow for 30 days (**Supplementary Table S1**), for the potted cotton in the sterilized diseased field soil, the group of cotton plants infected with *V. dahliae* showed the best in plant height, stem diameter, leaf age, and chlorophyll content, followed by the sterilized cotton plants group. The group with sterilized cotton stalks showed the worst performance in the treatments and the CK1 (cotton plants unreturned to the field). Potted cotton grown in the diseased field soil infected with *V. dahliae* also showed the same trend. However, the growth and development of cotton were better than those in the sterilized diseased field soil, especially as there were significant differences in the plant height and stem diameter indexes (*P<* 0.05). The preliminary results showed that the total amount of cotton plants that were returned to the field was beneficial for the growth of cotton.

Fifty days after the growth of potted cotton (**Supplementary Table S2**), the growth and development level of potted cotton in diseased soil was superior to that in sterilized soil, and the plant height index in seven treatments reached a significant difference level (*P<* 0.05). There were no significant differences in the plant height, the number of fruit branches, leaf age, and chlorophyll content of potted cotton in the treatments of the two potting substrates, respectively (*P >* 0.05); in the first fruiting branch height index, the cotton plants infected with *V. dahliae* group were significantly higher than those in the sterilized cotton stalks group (*P<* 0.05). The results showed that whether or not the potting substrate and the cotton plants were sterilized had an important effect on the growth and development of cotton.

When potted cotton grew to 70 days (**Table 2**), regardless of whether diseased or sterilized soil was used as the potting substrate or if cotton plants were directly returned to the field or not, there were no significant differences in the growth and development of cotton as compared with the CK (*P >* 0.05). The overall performance of the seven treatments was as follows: cotton plants infected with *V. dahliae* > sterilized cotton plants > CK > root stubble infected with *V. dahliae* > sterilized root stubble > cotton stalks infected with *V. dahliae* > sterilized cotton stalk. The results showed that the total return of cotton plants to the field promoted the growth and development of cotton to some extent. Whereas, the return of root stubble and cotton stalks to the field alone had a certain negative influence on the growth and development of cotton. The growth and development rate of potted cotton in sterilized soil was generally lower than that in corresponding treatments using diseased soil.

**Table 2:** Effect of the direct return to the field of cotton stalks and root stubble infected with *V. dahliae* on the growth and development of cotton (70th day).

Treatments	Plant height (cm)		Stems diameter (mm)		First fruiting branch height(cm)		Number of fruiting branches		Leaf age		Chlorophyll content (SPAD)	
	Sterilized diseased field soil	Diseased field soil infected with *V. dahliae*	Sterilized diseased field soil	Diseased field soil infected with *V. dahliae*	Sterilized diseased field soil	Diseased field soil infected with *V. dahliae*	Sterilized diseased field soil	Diseased field soil infected with *V. dahliae*	Sterilized diseased field soil	Diseased field soil infected with *V. dahliae*	Sterilized diseased field soil	Diseased field soil infected with *V. dahliae*
Sterilized cotton stalks	40.01 ± 5.17 a	73.92 ± 6.34 a *	12.01 ± 3.58 a	12.63 ± 1.08 a	17.89 ± 0.49 b	29.97 ± 1.48 a *	5.58 ± 0.67 a	6.00 ± 0.58 a	11.88 ± 0.74 a	14.29 ± 0.99 a	52.04 ± 1.65 ab	55.43 ± 0.44 a *
Sterilized root stubble	59.73 ± 5.27 a	73.71 ± 4.92 a	11.49 ± 0.94 a	12.72 ± 0.71 a	23.29 ± 0.75 ab	29.84 ± 0.86 a *	6.75 ± 0.72 a	6.29 ± 0.59 a	14.54 ± 0.6 a	14.13 ± 0.47 a	55.38 ± 1.18 a	55.53 ± 1.27 a
Sterilized cotton plants	60.00 ± 5.28 a	82.63 ± 5.15 a *	11.74 ± 1.15 a	13.85 ± 0.90 a	27.19 ± 1.02 a	31.78 ± 1.32 a *	5.5 ± 0.69 a	6.92 ± 0.63 a	13.5 ± 0.62 a	14.33 ± 0.75 a	52.37 ± 0.99 ab	55.14 ± 0.82 a
Cotton stalks infected with *V. dahliae*	45.81 ± 8.15 a	73.4 ± 6.72 a *	9.24 ± 1.59 a	12.45 ± 0.93 a	22.59 ± 1.43 a	31.93 ± 1.42 a *	4.27 ± 0.85 a	5.91 ± 0.67 a	12.05 ± 0.94 a	13.64 ± 0.74 a	49.08 ± 1.28 b	54.49 ± 1.19 a *
Root stubble infected with *V. dahliae*	45.16 ± 7.20 a	77.38 ± 5.33 a *	8.20 ± 1.37 a	13.23 ± 0.88 a *	22.02 ± 2.42 ab	33.92 ± 1.62 a *	4.25 ± 0.72 a	6.25 ± 0.48 a *	11.71 ± 0.72 a	14.42 ± 0.66 a *	51.76 ± 0.94 ab	54.9 ± 0.95 a *
Cotton plants infected with *V. dahliae*	57.2 ± 5.67 a	88.07 ± 7.96 a *	10.09 ± 1.29 a	15.42 ± 1.1 a *	28.68 ± 1.63 a	33.79 ± 1.99 a	5 ± 0.65 a	7.91 ± 0.77 a *	12.27 ± 0.63 a	14.95 ± 0.68 a *	54.66 ± 1.9 ab	61.05 ± 2.36 a
CK	54.60 ± 6.96 a	82.18 ± 4.35 a *	9.88 ± 1.17 a	13.5 ± 0.59 a *	19.46 ± 1.84 b	34.97 ± 4.00 a *	5.33 ± 0.75 a	7.13 ± 0.50 a	12.04 ± 0.72 a	14.21 ± 0.40 a *	54.84 ± 1.94 ab	55.68 ± 0.9 a

Data in the table are means ± standard errors; columns without the same letter are significantly different from each other (Tukey methods, significant level P< 0.05); and paired t-tests were performed for the seven treatments of sterilized and diseased field soils. Asterisks (*) indicate significant differences. The same is below.

### 3.2 Effects of directly returning cotton stalks and root stubble infected with *V. dahliae* to the field on the occurrence of cotton Verticillium wilt and the number of microsclerotia in rhizosphere soil

**Figure 1:**
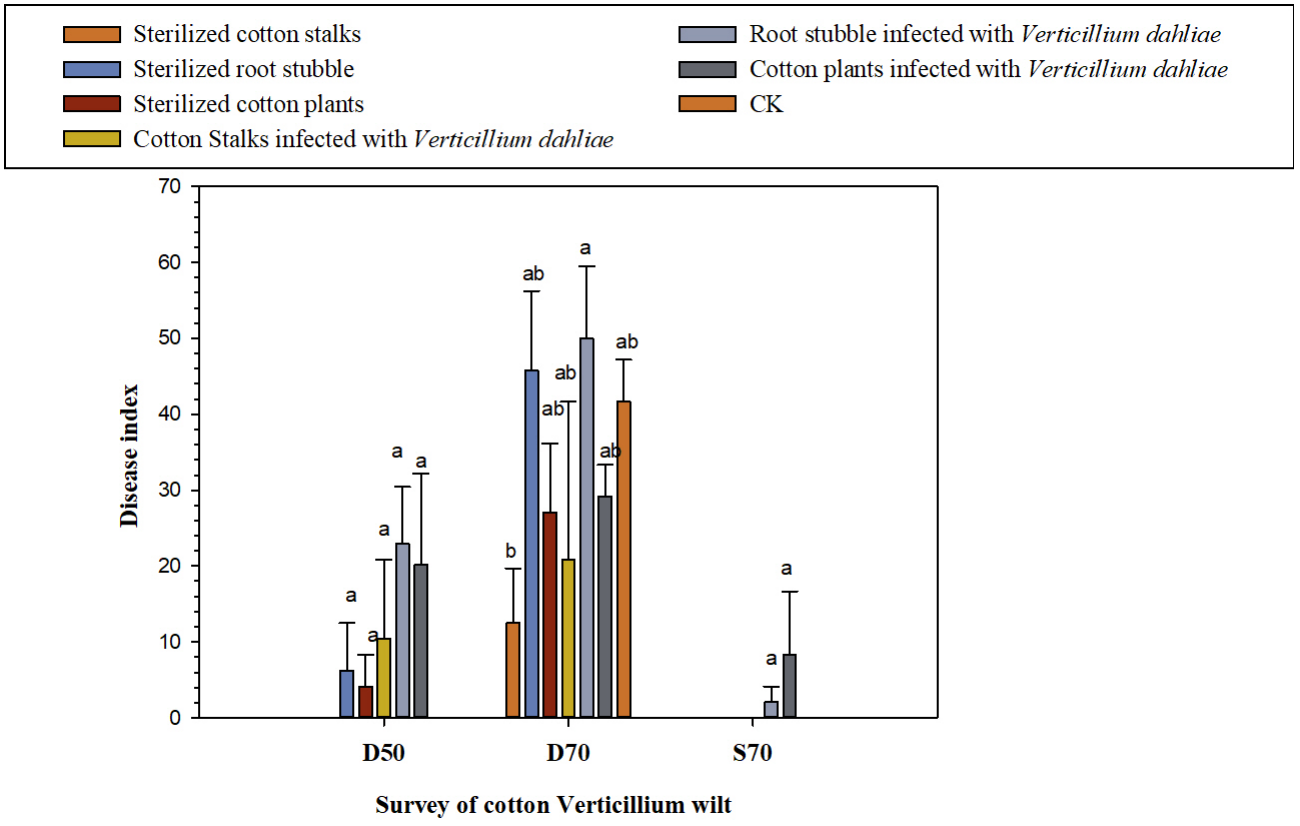
Effect of the direct return to the field of cotton stalks and root stubble infected with *V. dahliae* on the occurrence of cotton Verticillium wilt [D50 (70): the 50th (70th) day of the growing of cotton plants in diseased soil containing *V. dahliae*; S70: the 70th day of the growing of cotton plants in sterilized diseased field soil; at 50th day, no cotton Verticillium wilt occurred in all treatments].

According to **Figure 1** and **Table 3**, after 50 days of the potted experiment, when diseased field soil containing *V. dahliae* was used as the potting substrate, Verticillium wilt began to appear in cotton plants from all five treatments except for the sterilized cotton stalks group and control treatments, began to present Verticillium wilt. The disease index of the root stubble infected with *V. dahliae* group was the highest (22.92 ± 7.51), followed by cotton plants infected with *V. dahliae* group (20.14 ± 12.05). When potted cotton grew for 70 days, Verticillium wilt occurred in the seven treatments, and the disease index of the group with root stubble infected with *V. dahliae* group was the highest (50.00 ± 9.55), which increased by 20.00% as compared with the CK (41.67 ± 5.51). This was followed by the group with sterilized root stubble (45.83 ± 10.42), in which the disease index increased by 9.98% as compared with the CK. The disease index of the other four treatments was lower than that of CK, and the disease index of the sterilized cotton stalk treatment was the lowest. When sterilized soil was used as the potting substrate, Verticillium wilt occurred only in groups where both the cotton plants and root stubble were infected with *V. dahliae*. The disease index of the cotton plants infected with *V. dahliae* group (8.33 ± 8.33) was higher than that of the group where root stubble was infected with *V. dahliae* (2.08 ± 2.08), but the overall incidence remained low. Preliminary results indicated that the direct return of root stubble to the field had an aggravating effect on the occurrence of cotton Verticillium wilt in diseased fields, especially the direct return of root stubble infected with *V. dahliae*. The disease index of Verticillium wilt in potted cotton grown in diseased field soil was much higher than that for potted cotton grown in sterilized soil. The direct return of cotton plants infected with *V. dahliae* to the field aggravated the early onset of Verticillium wilt in potted cotton in diseased fields. The diseased soil had an essential effect on the occurrence of Verticillium wilt in cotton.

**Table 3:** Effect of the direct return of the cotton stalks and root stubble infected with *V. dahliae* to the diseased soil containing *V. dahliae* on the rate of increase or decrease in disease index of the cotton Verticillium wilt.

Treatments	Disease index of the D70	Rate of increase or decrease (%)
Sterilized cotton stalks	12.50 ± 7.22 b	−70.00
Sterilized root stubble	45.83 ± 10.42 ab	+9.98
Sterilized cotton plants	27.08 ± 9.08 ab	−35.01
Cotton Stalks infected with *V. dahliae*	20.83 ± 20.83 ab	−50.01
Root stubble infected with *V. dahliae*	50.00 ± 9.55 a	+20.00
Cotton plants infected with *V. dahliae*	29.17 ± 4.17 ab	−30.00
CK	41.67 ± 5.51 ab	

As can be seen from **Table 4**, the number of cotton Verticillium wilt microsclerotia in the diseased rhizosphere soil of potted cotton increased by varying degrees in each treatment as compared with the original soil sample. The number of microsclerotia in the group with root stubble infected with *V. dahliae* group (26.43 ± 5.51 Colony-Forming Units (CFU) g^−1^) was significantly higher than that in the CK (3.55 ± 1.21 CFU g^−1^)(*P*< 0.05). The cotton plants infected with *V. dahliae* group (14.80 ± 0.44 CFU g^−1^) and the group where cotton stalks were infected with *V. dahliae* (9.97 ± 3.41 CFU g^−1^) followed. The numbers of microsclerotia in the three treatments were 8.37, 4.25, and 2.54 times more than those in the original soil samples, respectively. The numbers of Verticillium wilt microsclerotia in the other three treatments were similar to those in the CK; they ranged from 3.26 CFU g^−1^ to 4.98 CFU g^−1^. In the seven treatments containing sterilized soil potting cotton, the microsclerotia of cotton Verticillium wilt were detected in the three treatments of additives with *V. dahliae*, and the number of cotton Verticillium microsclerotia was significantly higher in the group of cotton stalks infected with *V. dahliae* and the group of cotton plants with *V. dahliae* than the group of root stubble infected with *V. dahliae* (0.20 ± 0.20 CFU g^−1^) (*P<* 0.05). This could be attributed to the high amount of microsclerotia in the initial cotton stalk sample (11.38 ± 5.77 CFU g^−1^) and the low amount of microsclerotia in the initial root stubble sample (0.08 ± 0.03 CFU g^−1^), as well as the uneven distribution of microsclerotia in the rhizosphere soil. Overall, the direct return of cotton stalks and root stubble infected with *V. dahliae* increased both the risk and degree of Verticillium wilt occurrence. The disease index of potted cotton (**Table 3**) showed a positive correlation with the number of microsclerotia. However, despite having a low number of microsclerotia in the rhizosphere soil, sterilized root stubble resulted in a high disease index. The specific reasons behind these observations needed further exploration.

**Table 4:** Effect of the direct return cotton stalks and root stubble with *V. dahliae* to the field on the number of cotton Verticillium wilt microsclerotia in the cotton rhizosphere soil (DS70: the samples on the 70th day of the cotton rhizosphere soil in diseased soil containing *V. dahliae*; SS70: the samples of the 70th day of the cotton rhizosphere soil in sterilized diseased field soil).

Treatments		Number of cotton Verticillium wilt microsclerotia (CFU g^−1^)	Growth multiples
**DS70**	Cotton stalks infected with *V. dahliae*	9.97 ± 5.91 b	+2.54
	Root stubble infected with *V. dahliae*	26.43 ± 9.55 a	+8.37
	Cotton plants infected with *V. dahliae*	14.80 ± 0.76 ab	+4.25
	Sterilized cotton stalks	4.98 ± 1.63 b	+0.77
	Sterilized root stubble	3.26 ± 0.46 b	+0.16
	Sterilized cotton plants	3.67 ± 0.70 b	+0.30
	CK	3.55 ± 2.09 b	+0.26
	Soil samples during sowing	2.82 ± 0.42	
**SS70**	Cotton stalks infected with *V. dahliae*	5.02 ± 1.10 a	–
	Root stubble infected with *V. Dahliae*	0.20 ± 0.20 b	–
	Cotton plants infected with *V. dahliae*	3.21 ± 0.58 a	–
	Initial cotton stalks sample	11.38 ± 5.77	–
	Initial root stubble sample	0.08 ± 0.03	–

### 3.3 Transformation ability of insect–microorganism composite technology for the cotton stalks and root stubble infected with *V. dahliae* and risk assessment of this system to spread cotton Verticillium wilt

#### 3.3.1 Effects of microbial fermentation process on the pile temperature of the six materials

**Figure 2:**
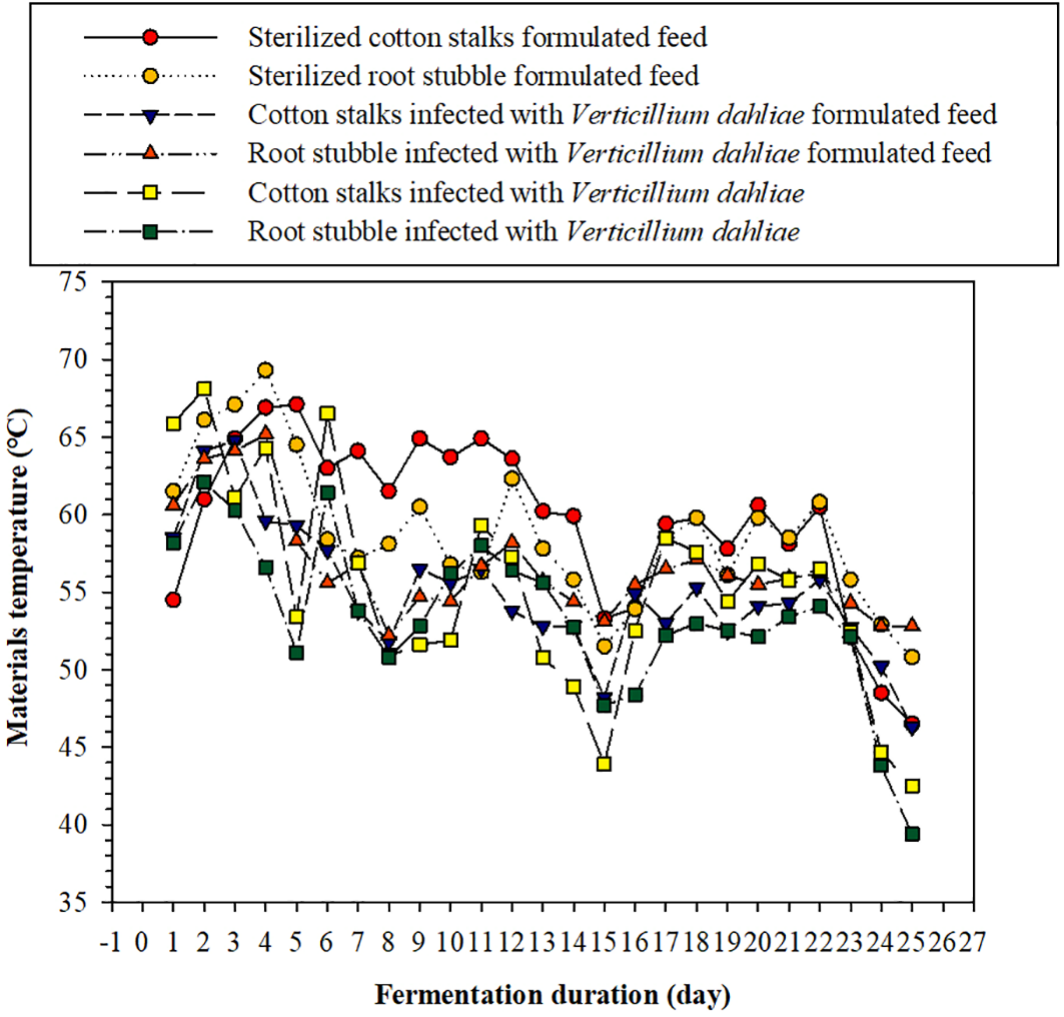
Effect of fermentation duration on the pile temperature of the six materials investigated here.

As can be seen from **Figure 2**, during the fermentation process, the temperature of each material pile initially increased and then fluctuatingly decreased; the temperature of each treatment was maintained above 50°C for the first 10 days, with a maximum temperature close to 70°C. After 22 days of fermentation, the temperature of each material pile decreased, whereas the temperature of the four compound feed piles decreased slowly and was still above 45°C. The temperature of the cotton stalks and root stubble infected with *V. dahliae* groups dropped to ~40°C. To reduce unnecessary testing costs, we measured the number of cotton Verticillium wilt microsclerotia in the six kinds of materials fermented for 5 days first. No microsclerotia of cotton Verticillium wilt were detected after 5 days of fermentation in any of these materials, indicating that the microbial fermentation process could easily kill the microsclerotia and thus reduce the risk of spreading cotton Verticillium wilt caused by the indirect return of cotton stalk to the field.

#### 3.3.2 Effects of microbial fermentation on the number of Verticillium wilt microsclerotia in six kinds of materials

**Table 5** presents the difference in the ability of the larvae of *P. brevitarsis* to transform six kinds of materials fermented for 25 days. Under suitable conditions, the larvae of *P. brevitarsis* had good transformation ability for all kinds of compound feeds, especially for the cotton stalks infected with *V. dahliae*. For every 1 unit of dry larvae gains, the larvae of *P. brevitarsis* could consume 27.47 times of this feed and produce 23.88 times of larvae dung-sand. The performance of root stubble infected with *V. dahliae* compound feed was the second; the ability to transform sterilized cotton stalks and sterilized root stubble compound feed ranked third. The ability of the larvae of *P. brevitarsis* to transform fermented pure cotton stalks and root stubble infected with *V. dahliae* was poor; among these, the root stubble infected with *V. dahliae* group was the worst, and the feed utilization rate was only 44.14% ± 0.75%. Cotton Verticillium wilt microsclerotia were not detected in larvae dung-sand formed from the six kinds of transformed materials. Therefore, there was no risk of spreading cotton Verticillium wilt when cotton stalks and root stubble compounds fermented for 25 days were converted into larvae dung-sand.

**Table 5:** Differences in the transforming capacity of the larvae of the *Protaetia brevitarsis* for six materials investigated here.

Processing treatments	Food intake (g)	Larvae weight gain (g)	Dung-sand weight (g)	Feed utilization rate (%)	Dung-sand conversion rate (%)	Mortality (%)
Sterilized cotton stalks compound feed	58.14 ± 0.38 a	1.95 ± 0.08 c	48.16 ± 0.66 b	72.67 ± 0.47 a	85.69 ± 0.78 a	2.00 ± 2.00 a
Sterilized root stubble compound feed	51.90 ± 0.42 b	2.08 ± 0.07 ab	40.07 ± 0.38 c	64.87 ± 0.52 b	80.42 ± 0.19 b	2.00 ± 2.00 a
Cotton stalks infected with *V. dahliae* compound feed	59.61 ± 0.58 a	2.17 ± 0.03 ab	51.81 ± 0.33 a	74.52 ± 0.72 a	90.21 ± 0.71 a	0.00 ± 0.00 a
Root stubble infected with *V. dahliae* compound feed	57.76 ± 0.59 a	2.25 ± 0.02 a	47.47 ± 0.37 b	72.21 ± 0.74 a	85.53 ± 1.00 a	0.00 ± 0.00 a
Cotton stalks infected with *V. dahliae*	40.03 ± 0.71 c	1.95 ± 0.09 c	32.59 ± 0.24 d	50.04 ± 0.88 c	85.68 ± 1.61 a	2.00 ± 2.00 a
Root stubble infected with *V. dahliae*	35.31 ± 0.60 d	1.89 ± 0.08 c	26.17 ± 0.20 e	44.14 ± 0.75 d	78.40 ± 1.62 b	2.00 ± 2.00 a

## 4 Discussion

### 4.1 Effects of the direct return of cotton stalks and root stubble infected with *Verticillium dahliae* to the field on cotton growth and development

Potted experiments were conducted in this study to investigate the effects of the direct return of cotton stalks and root stubble with *V. dahliae* on the growth and development of cotton and the occurrence of Verticillium wilt. The findings demonstrated that the total return of cotton plants to the field had a certain positive influence on the growth and development of cotton, which was consistent with the previous studies that had reported that returning stalks to the field could act as fertilizer for the subsequent seasons of cotton cultivation (Wang et al., 2014; Lu et al., 2022; Yan et al., 2022; Zhang et al., 2023a). However, this study also found that the return of cotton stalks and root stubble infected with *V. dahliae* to the field alone may have a particularly negative impact on the growth and development of cotton. This could be attributed to several factors, including the short return time of cotton plants to the field, the insufficient decomposition of organic matter by microorganisms, the autotoxicity resulting from the decomposition of the returned cotton plants, and the limited nitrogen availability leading to competition and comprises the fertilizer effect of returned plants (Tesio et al., 2012; Li and Zhang, 2016; Qin et al., 2022). As for the promotion of cotton growth and development by the total return of cotton plants to the field, it should be further verified whether root stubble can contribute to the decomposition of cotton stalks and vice versa. The direct return of cotton stalks and root stubble infected with *V. dahliae* to the field could also increase the number of microsclerotia in rhizosphere soil, thereby increasing the risk of Verticillium wilt in cotton and its negative effects (Wang et al., 2011; Wang et al., 2012; Liu et al., 2019). Contrarily, the growth rate of potted cotton with sterilized soil as potting substrate was generally lower than that of diseased soil as potting substrate. The growth and development of cotton in the three treatments infected with *V. dahliae* were also better than those in the three treatments supplemented with sterilized materials, indicating that both the microorganisms carried by cotton plants and soil microorganisms had important effects on the growth and development of cotton. While sterilized soil or materials killed the pathogen of cotton Verticillium wilt, they also eliminated other microorganisms beneficial to the growth and development of cotton (Wang et al., 2006; Yang et al., 2012; Kumar and Verma, 2018).

### 4.2 Effects of the direct return of cotton stalks and root stubble infected with *V. dahliae* to the field on the occurrence of cotton Verticillium wilt and the number of microsclerotia in rhizosphere soil

This study proved that the direct return of cotton stalks and root stubble that had been infected with *V. dahliae* increased both the risk and degree of Verticillium wilt occurrence in the ongoing year. The number of microsclerotia of cotton Verticillium in the rhizosphere soil of the affected field increased by varying degrees (0.16–8.37 times) as compared with the initial soil samples during the peak of Verticillium wilt occurrence. There was a positive correlation between the disease index of cotton Verticillium wilt occurrence and the number of microsclerotia in the rhizosphere soil. However, the incidence and degree of cotton Verticillium wilt were dictated by combinations of inoculation and the fertilizer effect of cotton stalks and root stubble infected with *V. dahliae* being returned to the field. Other factors involved here were the base number of Verticillium wilt microsclerotia in the rhizosphere soil and environmental factors. Therefore, the overall situation was somewhat complicated (Guo et al., 2012; Yu, 2012; Liu et al., 2021b). It should be noted that the return of root stubble to the field significantly aggravated the occurrence of cotton Verticillium wilt in diseased fields, especially the return of root stubble infected with *V. dahliae* had the most significant impact on the occurrence of cotton Verticillium wilt. In this case, the disease index increased by 20.00%, and the number of Verticillium wilt microsclerotia increased by 8.37 times, possibly due to the cotton roots being a central part of the pathogen infestation of Verticillium wilt. The return of root stubble to the field created favorable conditions (the microsclerotia are released into the soil as the root stubble decays) for the propagation and infection of Verticillium wilt, increased the number of Verticillium wilt microsclerotia in the rhizosphere soil, and increased the risk and disease index of Verticillium wilt in cotton affected fields. The relevant mechanisms must be studied further (Raaijmakers et al., 2010; Zhu et al., 2017).

### 4.3 Transformation ability of insect–microorganism composite technology on the cotton stalks and root stubble infected with *V. dahliae* and the risk assessment of this system to spread cotton Verticillium wilt

The results of this study showed that the larvae of *P. brevitarsis* have an outstanding transformation ability to ferment cotton stalks and root stubble materials. Under the optimal conditions, for every 1 unit of dry larvae gain, the larvae of *P. brevitarsis* could consume 27.47 times of this feed and produce 23.88 times of larvae dung-sand; the results were consistent with Zhang et al. (2022). According to our observations, the collectible amount of cotton plants was 6,000 kg hm^−2^. As per conservative estimates, each hectare of cotton plants could produce 3,000 kg of larvae dung-sand and 120 kg of dry larvae. The utilization of cotton stalk and root stubble infected with *V. dahliae* mediated by insect–microorganism composite technology has much potential for field applications (Zhang, 2022). To investigate the potential risk of cotton Verticillium wilt occurrence resulting from converting cotton stalks and root stubble infected with *V. dahliae* into larvae dung-sand, the effects of the fermentation process on the number of microsclerotia of cotton Verticillium wilt in cotton materials were investigated. After 5 days of fermentation, no microsclerotia of cotton Verticillium wilt were detected in all six fermentation materials, indicating that the high temperature continuously generated during the fermentation process effectively killed the microsclerotia of cotton Verticillium wilt. These observations are consistent with the previous results that reported the removal of microsclerotia of cotton Verticillium wilt when treated at 55°C for 6 h (Shang et al., 2013). Therefore, after 5 days of fermentation, none of the six fermentation materials studied here posed a risk of spreading the microsclerotia of cotton Verticillium wilt upon return to the field. In addition, we measured the number of microsclerotia in the larvae dung-sand obtained from the six fermentation materials after fermentation for 25 days; no microsclerotia were detected. Therefore, there is no risk of transmitting cotton Verticillium wilt when converting cotton stalks and root stubble infected with *V. dahliae* into larvae dung-sand. Larvae dung-sand is rich in nutrients and contains beneficial microorganisms, making it practical for application in horticultural crops (Li et al., 2019; Shi et al., 2021). In addition, it is friendly to the environment (Zhang et al., 2020). It has the advantage of potentially promoting the growth and development of cotton while reducing the occurrence of cotton Verticillium wilt. Therefore, it is worthwhile to carry out further relevant experiments on the return of larvae dung-sand to the field after the biotransformation of cotton plants infected with *V. dahliae* (Joung et al., 2022; Zhang et al., 2022).

## 5 Conclusions

The direct return of cotton stalk and root stubble infected with *V. dahliae* has no noticeable effect on the growth and development of cotton in the current year, which may aggravate the occurrence of Verticillium wilt and the accumulation of microsclerotia in rhizosphere soil. As a risk response plan, the indirect return of cotton plants infected with *V. dahliae* to the field by insect–microorganism composite technology is worthy of further exploration for green prevention and control of Verticillium wilt and the sustainable development of the cotton industry.

## Data availability statement

The raw data supporting the conclusions of this article will be made available by the authors, without undue reservation.

## Author contributions

Experimental design and optimization: DM and PJ. Methodology: GZ and SQ. Validation: HG and JY. Data curation: GZ and ZM. Writing—original draft preparation: GZ and ZM. Writing—review and editing: DM and GZ. Supervision: PJ. Project administration: DM, SQ, and PJ. All authors contributed to the article and approved the submitted version.
