# Supplementary material for: Investigating Verticillium wilt occurrence in cotton and its risk management by the direct return of cotton plants infected with Verticillium dahliae to the field
Source: Front Plant Sci. 2023 Nov 3;14:1220921. doi: 10.3389/fpls.2023.1220921 (PMC10654977; doi:10.3389/fpls.2023.1220921)
Supplement: Supplementary file 1 [file DataSheet_1.docx]

Supplementary Material

Investigating verticillium wilt occurrence in cotton and its risk management by the direct return of cotton plants infected with *Verticillium dahliae* to the field

Guangjie Zhang^1,2,3^, Zhuo Meng^1,2,3^, Hao Ge^1,2,3^, Jiali Yuan^1,2,3^, Song Qiang^1,2,3^, Ping'an Jiang^2*^, Deying Ma^1,2,3*^

^1^College of Agronomy, Xinjiang Agricultural University, 311 Nongda East Road, Urumqi, China.

^2^Engineering Research Centre of Cotton, Ministry of Education, Xinjiang Agricultural University, 311 Nongda East Road, Urumqi, China.

^3^Key Laboratory of the Pest Monitoring and Safety Control on Crop and Forest, Xinjiang Agricultural University, 311 Nongda East Road, Urumqi, China.

*** Correspondence:** Deying Ma, [mdyxnd@163.com;](mailto:mdyxnd@163.com;) Ping'an Jiang, jiang863863@sina.com

# Supplementary Tables

**TABLE S1** Effect of direct return to the field of cotton stalks and root stubble infected with *V. dahliae*

on the growth and development of cotton (30^th^ day)

| **Treatments** | **Plant height (cm)** | | **Stems diameter (mm)** | | **Leaf age** | | **Chlorophyll content (SPAD)** | |
| --- | --- | --- | --- | --- | --- | --- | --- | --- |
|  | **Sterilized diseased field soil** | **Diseased field soil**  **infected with**  ***V. dahliae*** | **Sterilized diseased field soil** | **Diseased field soil infected with**  ***V. dahliae*** | **Sterilized diseased field soil** | **Diseased field soil**  **infected with**  ***V. dahliae*** | **Sterilized diseased field soil** | **Diseased field soil**  **infected with**  ***V. dahliae*** |
| Sterilized cotton stalks | 14.98±0.66 b | 25.94±2.10 b * | 2.27±0.15 b | 4.63±0.38 a * | 4.75±0.31 a | 5.99±0.54 a | 41.22±0.76 b | 49.19±1.48 a * |
| Sterilized root stubble | 19.52±1.28 ab | 25.76±1.81 b * | 3.20±0.21 ab | 4.78±0.31 a * | 6.04±0.24 a | 5.66±0.54 a | 42.65±1.73 b | 51.77±1.62 a * |
| Sterilized cotton plants | 20.08±1.68 ab | 27.65±1.35 b * | 3.43±0.22 a | 5.37±0.30 a * | 5.17±0.32 a | 6.46±0.26 a * | 43.05±1.59 ab | 49.90±1.10 a * |
| Cotton Stalks infected with  *V. dahliae* | 16.87±1.74 ab | 24.78±1.63 b * | 2.97±0.27 ab | 4.59±0.22 a * | 5.14±0.42 a | 6.14±0.44 a | 45.27±1.68 ab | 50.65±1.14 a * |
| Root stubble infected with  *V. dahliae* | 19.28±1.79 ab | 28.70±1.14 ab * | 3.09±0.28 ab | 4.79±0.26 a * | 5.13±0.38 a | 6.79±0.30 a * | 49.08±1.55 a | 50.07±1.27 a |
| Cotton plants infected with  *V. dahliae* | 22.48±1.25 a | 34.54±1.66 a * | 3.41±0.17 a | 5.61±0.32 a * | 5.55±0.24 a | 7.18±0.35 a * | 46.37±1.40 ab | 52.27±1.30 a * |
| CK | 18.09±1.61 ab | 27.86±1.36 ab * | 2.98±0.31 ab | 4.90±0.24 a * | 5.00±0.35 a | 6.63±0.33 a * | 42.18±1.44 b | 52.10±1.26 a * |

Note: Data in the table are means ± standard errors; columns without the same letter are significantly different from each other (Tukey methods, significant level *P*<0.05); and paired t-tests were performed for the seven treatments of sterilized and diseased field soils. * Indicating significant differences. The same as below.

**TABLE S2** Effect of direct return to the field of cotton stalks and root stubble infected with *V. dahliae*

on the growth and development of cotton (50^th^ day)

| **Treatments** | **Plant height (cm)** | | **Stems diameter (mm)** | | **First fruiting branch height(cm)** | | **Number of fruiting branches** | | **Leaf age** | | | **Chlorophyll content (SPAD)** | | |
| --- | --- | --- | --- | --- | --- | --- | --- | --- | --- | --- | --- | --- | --- | --- |
|  | **Sterilized diseased field soil** | **Diseased field soil infected with**  ***V. dahliae*** | **Sterilized diseased field soil** | **Diseased field soil infected with**  ***V. dahliae*** | **Sterilized diseased field soil** | **Diseased field soil infected with**  ***V. dahliae*** | **Sterilized diseased field soil** | **Diseased field soil infected with**  ***V. dahliae*** | **Sterilized diseased field soil** | **Diseased field soil infected with**  ***V. dahliae*** | | **Sterilized diseased field soil** | | **Diseased field soil infected with**  ***V. dahliae*** |
| Sterilized cotton stalks | 26.46±2.25 a | 47.14±3.99 a * | 5.29±0.58 a | 9.68±0.73 ab * | 18.33±0.67 b | 26.53±1.30 b * | 1.46±0.53 a | 2.50±0.58 a | 9.29±0.52 a | | 11.12±0.75 a | 46.68±0.85 a | 52.23±1.12 a * | |
| Sterilized root stubble | 38.08±3.05 a | 48.33±3.50 a * | 7.54±0.70 a | 9.81±0.61 ab * | 22.68±1.24 ab | 27.86±0.55 ab * | 3.13±0.67 a | 3.00±0.55 a | 10.57±0.58 a | | 11.25±0.43 a | 49.27±0.80 a | 50.62±1.30 a | |
| Sterilized cotton plants | 38.15±3.58 a | 54.77±3.48 a * | 7.77±0.77 a | 11.32±0.73 ab * | 26.13±1.34 a | 29.60±0.87 ab * | 2.17±0.62 a | 3.71±0.55 a | 10.25±0.47 a | | 12.23±0.47 a * | 50.03±0.67 a | 52.42±1.24 a | |
| Cotton Stalks infected with  *V. dahliae* | 31.64±4.55 a | 48.00±3.65 a * | 6.50±1.08 a | 8.94±0.78 b | 21.95±1.14 ab | 29.23±1.00 ab * | 1.64±0.74 a | 2.86±0.52 a | 8.82±0.66 a | | 11.10±0.54 a * | 46.03±1.40 a | 51.60±0.79 a * | |
| Root stubble infected with  *V. dahliae* | 33.42±4.04 a | 51.71±3.68 a * | 6.12±0.89 a | 9.71±0.67 ab * | 24.02±1.05 a | 29.18±1.05 ab * | 1.50±0.60 a | 3.50±0.41 a * | 9.29±0.51 a | | 11.59±0.52 a * | 47.94±1.90 a | 53.14±1.66 a | |
| Cotton plants infected with  *V. dahliae* | 41.55±3.12 a | 61.91±4.98 a * | 7.19±0.70 a | 12.21±1.13 a * | 25.77±1.77 a | 31.15±1.49 a | 2.23±0.50 a | 4.64±0.54 a * | 9.86±0.40 a | | 12.29±0.46 a * | 51.26±2.57 a | 53.77±1.20 a | |
| CK | 33.88±4.31 a | 54.58±3.16 a * | 6.63±0.91 a | 10.4±0.47 ab * | 23.21±1.56 ab | 29.40±1.00 ab * | 1.71±0.52 a | 3.88±0.39 a * | 9.46±0.58 a | | 11.79±0.34 a * | 45.92±1.47 a | 51.27±1.30 a * | |
